# Ancient DNA Analysis of Mid-Holocene Individuals from the Northwest Coast of North America Reveals Different Evolutionary Paths for Mitogenomes

**DOI:** 10.1371/journal.pone.0066948

**Published:** 2013-07-03

**Authors:** Yinqiu Cui, John Lindo, Cris E. Hughes, Jesse W. Johnson, Alvaro G. Hernandez, Brian M. Kemp, Jian Ma, Ryan Cunningham, Barbara Petzelt, Joycellyn Mitchell, David Archer, Jerome S. Cybulski, Ripan S. Malhi

**Affiliations:** 1 Department of Anthropology, University of Illinois, Urbana, Ilinois, United States of America; 2 College of Life Science, Jilin University, Changchun, Jilin, China; 3 School of Integrative Biology, University of Illinois, Urbana, Ilinois, United States of America; 4 W.M. Keck Center for Comparative and Functional Genomics, University of Illinois, Urbana, Ilinois, United States of America; 5 Department of Anthropology and the School of Biological Sciences, Washington State University, Pullman, Washington, United States of America; 6 Department of Bioengineering, University of Illinois, Urbana, Ilinois, United States of America; 7 Institute for Genomic Biology, University of Illinois, Urbana, Ilinois, United States of America; 8 Department of Computer Science, University of Illinois, Urbana, Ilinois, United States of America; 9 Metlakatla Treaty Office, Metlakatla, British Columbia, Canada; 10 Northwest Community College, Prince Rupert, British Columbia, Canada; 11 Canadian Museum of Civilization, Gatineau, Quebec, Canada; Natural History Museum of Denmark, Denmark

## Abstract

To gain a better understanding of North American population history, complete mitochondrial genomes (mitogenomes) were generated from four ancient and three living individuals of the northern Northwest Coast of North America, specifically the north coast of British Columbia, Canada, current home to the indigenous Tsimshian, Haida, and Nisga’a. The mitogenomes of all individuals were previously unknown and assigned to new sub-haplogroup designations D4h3a7, A2ag and A2ah. The analysis of mitogenomes allows for more detailed analyses of presumed ancestor–descendant relationships than sequencing only the HVSI region of the mitochondrial genome, a more traditional approach in local population studies. The results of this study provide contrasting examples of the evolution of Native American mitogenomes. Those belonging to sub-haplogroups A2ag and A2ah exhibit temporal continuity in this region for 5000 years up until the present day. Of possible associative significance is that archaeologically identified house structures in this region maintain similar characteristics for this same period of time, demonstrating cultural continuity in residence patterns. The individual dated to 6000 years before present (BP) exhibited a mitogenome belonging to sub-haplogroup D4h3a. This sub-haplogroup was earlier identified in the same general area at 10300 years BP on Prince of Wales Island, Alaska, and may have gone extinct, as it has not been observed in any living individuals of the Northwest Coast. The presented case studies demonstrate the different evolutionary paths of mitogenomes over time on the Northwest Coast.

## Introduction

The majority of studies investigating mitochondrial genomes (mitogenomes) [1]–[5], high-resolution Y-chromosomes [6]–[7], and genome-wide autosomal data [8]–[9] of Native Americas aim to reconstruct the evolutionary history and infer past demographic events of these populations (recently reviewed by [10]). The geographical distributions and frequencies of DNA variants exhibited by contemporary populations in the Americas are usually explained as the result of independent population migrations or significant regional demographic events (such as population bottlenecks, expansions, admixture, population structure) or a combination of these circumstances [5], [9]. However, a relatively high percentage of Y-chromosomes of Native Americans can be traced back to a European origin as a result of admixture following European contact [6], [11]–[13]. Moreover, genome-wide autosomal data of Native Americans usually exhibit significant amounts of European ancestry [8]–[9]. In addition to non-indigenous admixture, European colonization complicates the genomic analyses of living populations because large-scale population loss, movements and amalgamations of indigenous peoples occurred after European contact [14]–[16], making it difficult to render an understanding of pre-European contact population dynamics.

The genetic analysis of pre-European contact Native American remains can help researchers learn more about the early evolutionary history of Native Americans and circumvent bias in the dataset resulting from the effects European colonization [17]–[18]. The vast majority of ancient DNA samples analyzed in the Americas include individuals and populations from only the past three thousand years [17]. Expanding on the ideas and results originally presented in O'Rourke et al. [19], Raff et al. [17] performed an analysis that suggests that mitochondrial DNA haplogroups in the Americas exhibit temporal stability in haplogroup frequencies over the past few thousand years. A relatively small number of human skeletal remains have been identified in the Americas that are in excess of 5000 years old (the mid-Holocene) [20]. Of these, six have yielded analyzable DNA and were published in peer review journals [21]–[25]. An ancient individual dating to 10300 years before present (BP) from On Your Knees Cave on Prince of Wales Island in Southeast Alaska was analyzed for nearly the entire control region of the mitochondrial genome plus diagnostic sites of the coding region of the mitochondrial genome that assisted in the determination of his mitochondrial genome belonging to sub-haplogroup D4h3a [23]. DNA was extremely well preserved in the molars of this individual, permitting sex identification as male and determination that he belongs to the Q1a3a1a branch of the human Y-chromosome tree. The mitochondrial DNA of the five remaining individuals was characterized by sequencing portions of the hypervariable region and/or screening coding region markers definitive of Native American haplogroups. Of these, an individual uncovered in western Nevada with a radiocarbon date of 9200+/−60 years BP belongs to mitochondrial haplogroup C [22]. Haplogroup B was identified for an individual found in Hourglass Cave in Colorado who died approximately 8000 years BP [21]. Haplogroup A was determined for an individual uncovered at Big Bar Lake, British Columbia with a radiocarbon date of 4975+/−40 years BP [24]–[25]. Two individuals from China Lake, British Columbia, found in the same burial with a radiocarbon date of 4950+/−170 years BP were determined to belong to a form of macrohaplogroup M that has yet to be identified in any extant Native American population [24], [26]. The China Lake study suggests that individuals in the early to mid-Holocene may exhibit mitogenomes that have since gone extinct in a specific geographic region or in all of the Americas. In addition to the skeletal remains, 31 coprolites from Paisley Caves, Oregon, with dates ranging from ∼12300–1,300 years BP were assayed for diagnostic coding region mitochondrial DNA substitutions and placed into haplogroups A (68%) and B (32%) based on these assays [27]–[28]. As most populations in this geographic region today exhibit high frequencies of haplogroups B and D [29], the composite haplogroup frequency distribution for Paisley Caves is different and suggests temporal instability in mitochondrial genomes for this geographic region commonly referred to as the Columbia Plateau.

Sequencing mitogenomes in living groups provides a higher resolution of maternal relationships in populations in Northeast Asia and the Americas [1]–[4]. Importantly, with recent advances in biotechnologies it is now possible to obtain complete mitochondrial genomes from ancient individuals [27], [30]–[31]. The combination of ancient mitogenomes and accurate radiocarbon dates from ancient individuals also provides an independent source of information to test hypotheses of rates of molecular clocks [23]. To date, the only complete mitochondrial genome from an ancient Native American individual comes from an archaeological site in Greenland with a radiocarbon date of approximately 4000 years BP [27].

**Figure 1:**
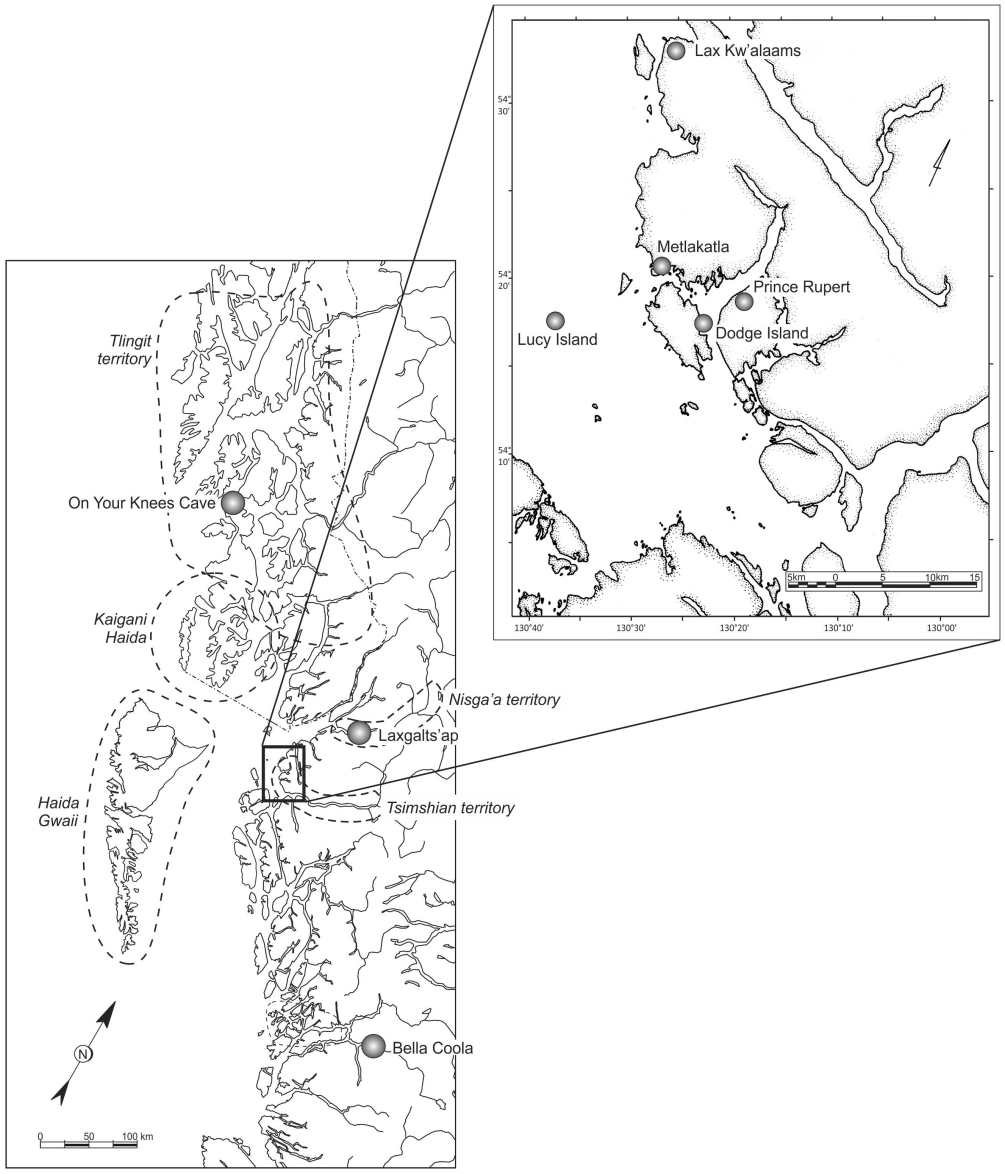
Map of study area.

To increase our understanding of mitochondrial DNA variation in the mid-Holocene of North America, we sequenced mitogenomes of skeletal remains from the coast of northern British Columbia, Canada, including the offshore Lucy Islands and the inner Prince Rupert Harbour region (Figure 1). The study provides examples of previously unknown North American lineages that exhibit at least 5000 years of temporal continuity but also a long-standing lineage that likely went extinct on the northern Northwest Coast soon after 5000 BP. These mitogenomes begin to provide a glimpse of mitogenomic diversity during mid-Holocene times in the Americas.

### Biocultural Context of Living and Ancient Individuals

The Lax Kw’alaams and Metlakatla are Tsimshian-speaking communities located in the Prince Rupert region of British Columbia. In 1862, a missionary, William Duncan, split the Lax Kw’alaams community to form the Metlakatla community. RSM, JSC and DA established a collaborative DNA study with these two communities in 2008. Additionally, the Laxgalts’ap is a Nisga’a-speaking community that resides along the Nass River directly northeast of the Prince Rupert region. RSM and JSC established a collaborative DNA study with the Laxgalts’ap in 2007. RSM, JSC and DA visit communities annually to collect saliva samples for DNA analysis and report to the communities on the most recent DNA results. The three mitochondrial genomes from living individuals generated in this study come from these three communities. A fourth mitochondrial genome from a living individual is from the Kaigani Haida and was previously published [7].

The Lucy Islands are an isolated cluster in Chatham Sound 19 km west of the city of Prince Rupert and its inner harbour (Figure 1). Traditionally, the Lucy Islands are included in the territory of the Gitwilgyots, a Tsimshian-speaking tribe that wintered in the Prince Rupert area at the time of European contact [32]. In late spring, during the seasonal round, the Gitwilgyots moved to the outer islands west of Prince Rupert for a period of marine fishing, shellfish gathering and sea mammal hunting before returning to the Skeena River in early summer for the salmon runs [33].

On the largest island, a small rectangular house depression adjacent to a large shell midden site (archaeological site GbTp-1) is inferred to be a seasonal camp [34]. Seven radiocarbon assays date the cultural deposits at this site from cal BP 7550 to cal BP 5280 [34]. The older dates are supported by the elevation of the cultural deposits above the shoreline. A sea level curve, created for neighboring islands, demonstrates that sea levels in the period between 8000 and 5000 radiocarbon years BP were higher than they are today [35]. The rectangular house depression adjacent to a shell midden is the earliest example of a pattern that became much more common in the Prince Rupert area between 2500 and 1500 years BP. The house depression at GbTp-1 is significant because it suggests that by 5000 years BP, the people of this area had large structures at seasonal camps that were likely occupied for only a few weeks out of the year [34]. The pattern of a rectangular house depression adjacent to a shell midden also suggests continuity in cultural practices from 5000 years BP to historic times.

The human remains we tested were found exposed on the shell midden deposit of GbTp-1 during the winter of 1984–1985 when two trees were felled by strong winds. The human remains were collected by personnel from the Museum of Northern British Columbia, Prince Rupert, and sent for osteological analysis to the Canadian Museum of Civilization, Gatineau. A brief report was filed [36] and the remains were assigned catalog numbers and accessioned by the latter institution with the approval of the Metlakatla Indian Band. The human remains included skull and jaw fragments, and an associated tibia of a young adult female (catalog XVII-B-938), upper and lower jaw parts of a small child (XVII-B-940), and an incomplete lower jaw of a late middle-aged or older male (XVII-B-939). The skull of the female included *cribra orbitalia,* a porousness in the roof of the eye socket possibly related to iron-deficiency anemia (see [37] for details concerning other Northwest Coast occurrences), and unusually thick, though otherwise normally appearing vault bones (the frontal bone ranged from 10 to 12 mm thick and the left parietal from 10 to 13 mm). The male jaw included greatly worn teeth, some with heavy deposits of calculus (tartar), alveolar abscess lesions at three tooth sites, and evidence for loss before death of three, and possibly all four incisors. The maximum length of the female tibia indicated an estimated living stature of 154.9 cm, or 5′1′ according to the method of Trotter and Gleser [38] for White females deemed appropriate for application to aboriginal Northwest Coast female skeletal remains (see [36]).

Measured collagen based radiocarbon ages of 5330±40 BP and 5710±40 BP were obtained for XVII-B-938 and XVII-B-939 respectively (Beta-294715 and Beta-317343). Conventional ages of 5530±40 BP and 5930±40 BP were also reported by Beta Analytic Inc. along with δ^13^C values of −12.9 and −11.6. Those values indicate a diet high in marine protein according to a scale developed for Prince Rupert Harbour skeletal remains [39] (see also [40]–[41]), and require compensation for a marine reservoir influence on radiocarbon age estimates from human bone. Application to the Lucy Island conventional ages based on arguments advanced elsewhere [40] provided two-sigma age ranges of cal BP 5870 to 5480 and cal BP 6260 to 5890 for XVII-B-938 and XVII-B-939 respectively.

We also tested two individuals from the inner harbor site of Dodge Island (GbTo-18) who were excavated with other human burials in 1967 as part of a North Coast Prehistory Project sponsored by the Canadian Museum of Civilization [42]. Like Lucy Island, the Dodge Island and other inner harbor sites excavated during the North Coast Prehistory Project consisted of shell deposits indicating variably long periods of intermittent occupation and use as cemeteries. Charcoal samples collected from Dodge Island provided radiocarbon dates of occupation from cal BP 5500 to cal BP 1800 [40].

Most of the human burials at this site (n = 20), as directly dated, were clustered in the period cal BP 2800 to cal BP 1800 [40]. Included was XVII-B-152 which yielded a direct (non-AMS) measured age of 2830+/−75 BP (S-1426) or cal BP 2770 to 2300 when corrected for the marine reservoir effect. The skeleton was that of a young adult female, most of which was recorded *in situ* as disturbed but probably flexed.

XVII-B-160a is a partially preserved disturbed skeleton from the same excavation level as a nearby charcoal sample dated at 4130 BP. A collagen sample from the skeleton provided a measured radiocarbon age of 4680+/−40 BP (Beta-202019). When calibrated and corrected for the marine reservoir effect, the two-sigma age range was cal BP 5130 to 4580. For the remainder of the article XVII-B-939, XVII-B-938, XVII-B-152 and XVII-B-160a will be referred to as Ancient 939, Ancient 938, Ancient 152 and Ancient 160a, respectively.

## Results

Full coverage of the ancient mitogenomes was obtained for all four samples. The average read length, read depth and total base pairs aligned to the mitogenome for the ancient samples are given in Table 1. The average gDNA fragment length is consistent with the expectations of degraded ancient DNA. The average quality score for all Illumina sequence reads was over 30 indicating high quality data with an inferred base call accuracy of 99.9%. Table 1 also provides information on the mitogenome haplogroup affiliation of the four ancient samples analyzed in this study.

**Table 1:** Ancient mitogenome analysis information.

Sample	Average Read Depth	Total BP Aligned to Reference mtDNA after Duplicate Removal	Average Read Length of Trimmed Reads	Mitochondrial haplogroup
152	58	965772	64	A2ag
160a	151	2502434	68	A2ah
938	62	1032311	80	A2ag
939	123	2045251	83	D4h3a7

The DNA patterns from the ancient samples do not exhibit cytosine deaminations in the beginning of a sequence read as a result of using a proof-reading high fidelity polymerase. However, the DNA fragmentation pattern exhibits an excess of purines at the genomic position preceding the read start (Figure S1). All DNA extraction and PCR blanks associated with the four ancient samples did not exhibit any amplification indicating that exogenous contamination was minimized in the analysis.

**Figure 2:**
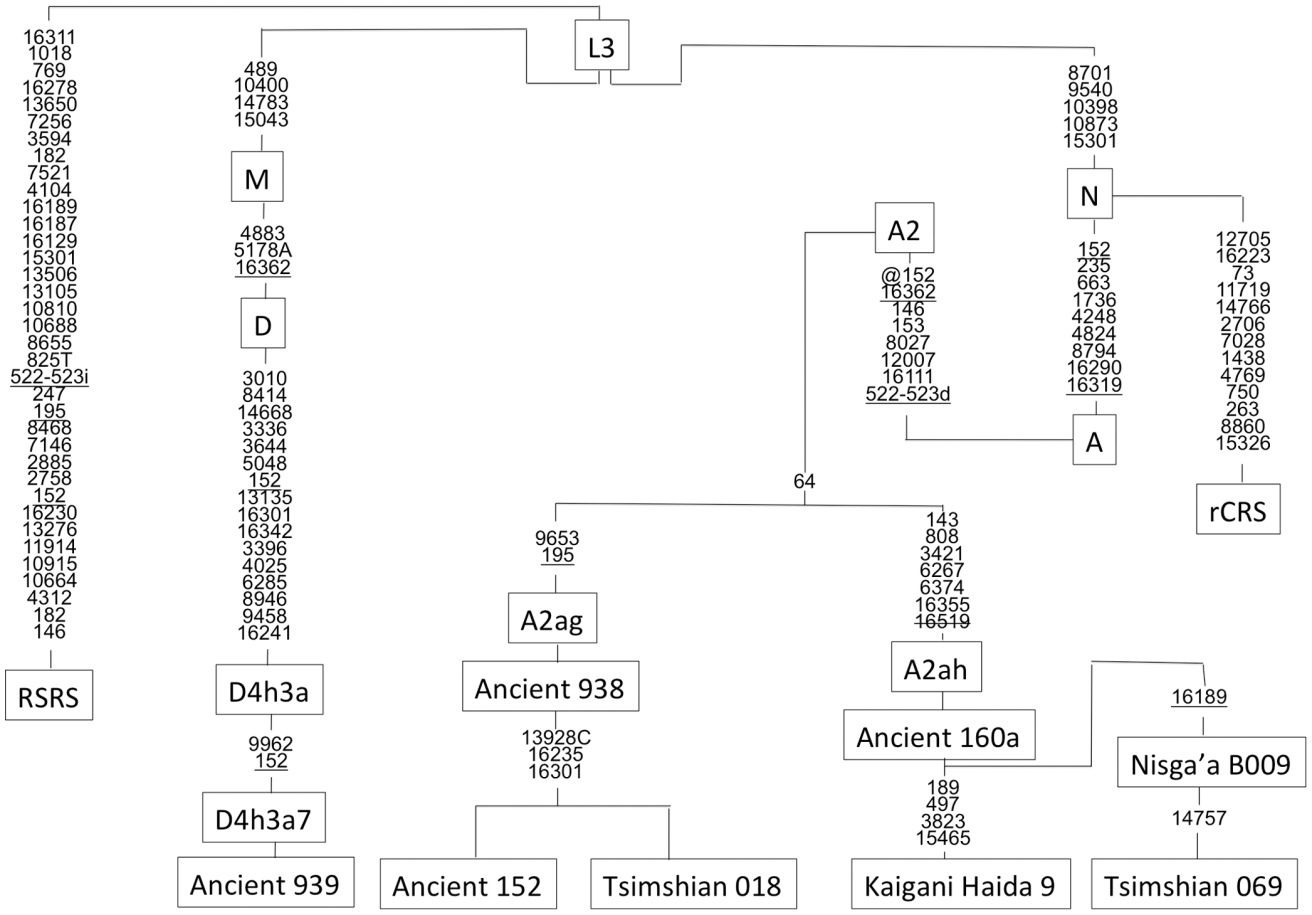
Phylogeny of complete mitochondrial genomes sequenced in this study. Mutations are transitions unless specified. Transversions are indicated by an A, G, C, or T after the nucleotide position. Insertions are indicated by an “i”, deletions are indicated by a “d”, recurrent mutations are underlined, and mutations back to the rCRS nucleotide are designated by a “@”. The C stretch length polymorphism in region 303–315 was disregarded in the tree. The sample “Haida 9″ was analyzed in Schurr et al. (2012). All other samples were analyzed in this study.

Ancient 939 is assigned to haplogroup D4h3a based on variants in the mitochondrial genome. This sample has unique transition substitutions at nucleotide positions (nps) 9962 and 152 (Figure 2). The same haplogroup and substitutions (via RFLP and HVSI sequence) were confirmed by the ancient DNA laboratory at Jilin University.

Ancient 938, Ancient 152 and Tsimshian 018 (the latter a living Tsimshian speaker from the Prince Rupert area) are assigned to a subclade of haplogroup A2 (Figure 2). This subclade is defined by substitutions at nucleotide positions 195 and 9653. Ancient 152 and Tsimshian 018 also share substitutions at a transversion at 13928 and transitions at 16235 and 16301. The haplogroups for Ancient 938 and Ancient 152 were confirmed from multiple extractions in the MMAL and Ancient 938 was confirmed from multiple extractions at Jilin University. The mitogenome sequenced from Ancient 938 at approximately 5500 years BP is related to the mitogenome sequenced from Ancient 152 at approximately 2500 years BP. Both ancient mitogenomes are similar or match a living Tsimshian-speaking individual Tsmishian 018.

Ancient 160a, Nisga’a B009 (a living Nisga’a individual), Tsimshian 069 (a living Tsimshian individual) and Kaigani Haida haplotype 9 [7] are assigned to a different subclade of haplogroup A2 (Figure 2). This subclade is defined by multiple transition substitutions at nps 143, 808, 3421, 6267, 6374, 16355 and 16519. Nisga’a B009 and Tsimshian 069 individuals have an additional substitution at np 16189. Although it should be noted that np 16189 is a hypervariable position and should not be used to define a subclade. Schurr et al. [7] reported a Kaigani Haida individual (haplotype 9) that belongs to this subclade and has additional substitutions at nps 189, 497, 3823 and 15465. Of the 41 Haida mitochondrial DNAs reported by Ward et al. [43], ten indviduals exhibit haplotypes (spanning nps 16024–16383) that match Ancient 160a with an additional two that are derived by transitions at nps 16188 and 16301, respectively. Moreover, six of 40 Bella Coola individuals match the HVSI of the Nisga’a B009 and Tsimshian 069 individuals [43].

In addition to the steps taken to confirm all of the ancient DNA results in this study, we note that none of the laboratory personnel in the MMAL and the ancient DNA lab at Jilin University belong to these mitochondrial haplogroups. These observations maximize our confidence in the authenticity of these results.

## Discussion

The presence of a house depression adjacent to a shell midden that contains human skeletal remains on Lucy Island is an early example of a feature that is repeated elsewhere in the Prince Rupert Harbour area almost to the time of historic European contact [34]. This suggests that the subsistence and residential patterns in this region have remained relatively stable for more than 5000 years. In this context, we discuss examples of the evolution of mitogenomes that are drastically different.

First, the mitochondrial genome of individual Ancient 939 exhibits substitutions that place it in sub-haplogroup D4h3a. This mitogenome exhibits two unique substitutions at nps 152 and 9962 and we designate it as sub-haplogroup D4h3a7 (Figure 2). Sub-haplogroup D4h3a is not identified in any ancient or living individuals on the Northwest Coast after approximately 6000 years BP [7], [29], [43], however, this haplogroup was previously identified in a 10300 years BP individual from On Your Knees Cave on Prince of Wales Island in Southeast Alaska [23]. This suggests that sub-haplogroup D4h3a is either in extremely low frequency or has gone extinct in living populations of the Northwest Coast, given that today D4h3a is found in approximately 1.5% of individuals throughout the Americas [23]. The observation that two of seven (29%) early-mid Holocene skeletal remains from different archaeological sites continent-wide exhibit mitochondrial sub-haplogroup D4h3a suggests that this sub-haplogroup may have been more frequent earlier in time. The absence of sub-haplogroup D4h3a on the Northwest Coast from present-day to just before 6,000 years ago could be the result of random genetic drift *in situ* and/or the result of population movements into the geographic region as multiple population expansions have been hypothesized in this geographic region during the middle Holocene [6]. This same pattern of the loss of genetic diversity was also observed near China Lake, British Columbia, where two individuals, dating to approximately 5000 years BP, exhibited a haplogroup not present in the living population of the same region [24], [26].

The Ancient 938 individual exhibits substitutions that place it in sub-haplogroup A2. However, the mitogenome of this individual also exhibits substitutions at np 195 and 9653. Therefore we assign the mitogenome of Ancient 938 to sub-haplogroup A2ag (Figure 2). Ancient 152, an individual dated to approximately 2500 years BP from Dodge Island in the Prince Rupert area, and Tsimshian 018, a living Tsimshian-speaking individual from the Prince Rupert area also exhibit substitutions at nps 195 and 9653 and, thus, also belong to sub-haplogroup A2ag. In addition, Ancient 152 and Tsimshian 018 exhibit a transversion at np 13928 and transitions at nps 16235 and 16301 that are not found in the mitogenome of Ancient 938.

The Ancient 160a individual, also dating to approximately 5000 years before present (4680+/−40 BP), exhibits substitutions that place it in sub-haplogroup A2. This ancient individual along with three other living individuals exhibit additional transition substitutions at np 143, 808, 3421, 6267, 6374, 16355 and 16519. Based on these additional substitutions we assigned the ancient individual and the three living individuals to sub-haplogroup A2ah. Overall, the mitogenomes of sub-haplogroup A2ag and A2ah demonstrate temporal continuity in the Prince Rupert and neighboring regions from the present to approximately 5000 years BP. While caution should be employed when considering matches using only the HVSI region of the mitochondrial genome because its fast mutation rate can lead to similarities due to homoplasy instead of shared recent ancestry, this sub-haplogroup has been previously reported at a frequency of ∼29% (12/41) among the Haida and 15% (6/40) among the Bella Coola [43]. The temporal continuity of mitogenomes in sub-haplogroups A2ag and A2ah fits with the archaeological patterns of housing structures that suggest temporal continuity of culture from the mid-Holocene to historic times [34].

The study significantly increases the number of mitogenomes analyzed from the mid-Holocene in the Americas and also identifies previously unknown mitogenomic diversity from British Columbia First Nation groups. We provide clear case studies of mitogenomes that exhibit different evolutionary paths over time. Mitogenomes belonging to sub-haplogroups A2ag and A2ah exhibit continuity through time in Northwest North America, whereas the mitogenome belonging to sub-haplogroup D4h3a is either in extremely low frequency or has gone extinct in this region. This study also shows the utility of using mitogenomes to provide detailed examples of maternal relationships through time and can potentially be used to calibrate molecular clock rates [23]. Additional genomic information from ancient individuals and populations throughout the Americas will provide needed insight into the evolutionary process and population history of Native Americans.

## Materials and Methods

DNA analysis was performed on individuals Ancient 938 and Ancient 939 from Lucy Island. In addition, Ancient 160a and Ancient 152 from the Dodge Island shell midden site (GbTo-18) in the inner Harbour region were also analyzed and included in this study. Lastly, DNA was extracted from the saliva of two living Tsimshian-speaking individuals from the Prince Rupert area and one Nisga’a-speaking individual from the Nass River using Oragene DNA Collection Kits (OGR-500). Prior to the analysis of ancient individuals, the Tsimshian and Nisga’a individuals were sequenced for the HVSI region of the mitochondrial genome. Following the DNA analysis of the ancient remains, the complete mitogenome of the three living individuals were sequenced as in Malhi et al. [24]. All mitogenomes were submitted to Genbank and were assigned accession numbers KC998701-KC998707.

### Ethics Statement

Permissions were obtained from the Canadian Museum of Civilization, the Metlakatla and the Lax Kw’alaams communities for destructive analysis of samples from the ancient individuals analyzed in this study. These samples were donated to the Malhi Molecular Anthropology Laboratory. Following the analysis, any remaining materials from the ancient individuals were returned to the Canadian Museum of Civilization.

Participants of this study provided written informed consent. The University of Illinois Urbana-Champaign Institutional Review Board approved the consent process and is documented in the University of Illinois IRB protocol # 10538. In addition, RSM and JSC visit the participants annually to provide the latest updates on the research study and answer any questions the participants may have.

### Ancient Sample Preparation and DNA Extraction

All sample preparations and DNA extractions were completed in an ancient DNA laboratory facility. A description of the facility is given below. DNA was extracted from a tooth for each individual. Surface contamination from each tooth was removed by submerging each tooth in 6% sodium hypochlorite (full strength Clorox bleach) for 6 minutes. The bleach was removed and all samples were then rinsed twice with DNA-free ddH_2_O and once with isopropanol to remove any remaining bleach (modification of [44]). Tooth samples were then placed in a UV crosslinker until dry. Approximately 0.20 grams of tooth powder was obtained for each DNA extraction using a dremmel tool at low speeds to minimize the production of heat. The tooth powder was then incubated in 4 ml of demineralization/lysis buffer (0.5 M EDTA, 33.3 mg/ml Proteinase K, 10% N-lauryl sarcosine) for 12–24 hours at 37°C. The digested sample was then concentrated to approximately 100 µl using Amicon centrifugal filter units. Following concentration, the digest was run through silica columns using the Qiagen PCR Purification Kit and eluted in 60 µl volume of DNA extract.

### Preparation of Genomic Library, Mitochondrial DNA Enrichment and Illumina Sequencing

Approximately 50 µl of DNA extract was used to create a genomic library with adapters that contained a unique index for each library. The following modifications were made to the TruSeq DNA Sample Preparation V2 protocol. The DNA extract was not sheared as the DNA is expected to be fragmented due to taphonomic processes. A 1∶20 dilution of adapters was used, as the DNA concentration in the extract is presumably low. Multiple Ampure Bead XP clean ups were completed in an attempt to remove any adapter-dimer that may have developed. A PCR amplification of the genomic library was prepped in the ancient DNA laboratory (25 µl reaction with 10 µM primers, 5x PCR Buffer, 10 mM Kapa DNTPs, KapaHiFi polymerase, genomic library) and then transported to thermocyclers in the contemporary laboratory, across campus, in a sealed environment. The KapaHiFi polymerase was used to amplify the libraries, as this enzyme has proof-reading properties similar to other polymerases that limit nucleotide misincorporations resulting from cytosine deamination [45]–[46]. Genomic libraries were amplified for 15–18 cycles, and were then cleaned with the Qiagen MinElute Purification Kit. The quality of the libraries were assessed on the Agilent 2100 Bioanalyzer using the High Sensitivity DNA kit.

Cleaned libraries were then divided into aliquots of 5 µl to use in additional amplifications until the final concentration of all pooled libraries reached 100 ng/µl. A target enrichment of the mitochondrial genome was then performed on the amplified library using a Rivia customized target enrichment kit following the Rivia target enrichment protocol. A final post-enrichment amplification was performed for 15 cycles. The post-enrichment amplified product was then quantified using qPCR and submitted to High-Throughput Sequencing Division of the W.M. Keck Biotechnology Center at the University of Illinois Urbana-Champaign.

### Bioinformatics Analysis

Raw data from the Illumina HiSeq 2000 platform was analyzed with CASAVA 1.8.2. In order to limit contamination that may have been introduced after the clean room library-building step, any reads that did not exhibit the exact index sequence were discarded. Adapter sequences were trimmed using AdapterRemoval [47] with a minmum length of 25. Sequence reads were mapped to the human mitochondrial genome Cambridge reference sequence using the Burrows-Wheeler Aligner (BWA) 0.6.2 [48] with default parameters except for seed length, which was set to 1000. Duplicate reads were filtered based on mapping positions (-rmdup -s) using the SAMtools package 0.1.18 [49]. SNPs and INDELs were called using the SNVer package 0.4.1 [50]. SNP quality thresholds were set with a haploid model, a read depth of 20, and base quality of 20.

DNA damage (type I and type II) was assessed by comparing T –>C/G–>A and C–>T/A –>G transitions, respectively. A specific pattern of DNA damage has been identified in other ancient DNA studies [30], [46] [51]. These studies show a pattern of increased type II DNA damage at the beginning and end of degraded DNA fragments. An additional pattern can be inferred from an excess of purines at the genomic position before the sequencing start, which is indicative of strand fragmentation subsequent to post-mortem depurination [46]. We compared our results to other studies to assess if we see similar patterns of DNA damage.

### Contamination Control and Independent Replication

All sample preparations, DNA extractions and PCR amplification setups were completed in the ancient DNA laboratory facility at the University of Illinois. The ancient DNA lab is a positively pressured clean room with hepa-filtered air. The clean room contains an anteroom and air flows from the ancient DNA lab to the anteroom to the hallway. Personnel working in the ancient DNA lab wear disposable hairnets, facemasks, laboratory coveralls and booties. All equipment, reagents and consumables are dedicated for use in the ancient DNA laboratory. The ancient DNA lab is routinely cleaned with bleach and all containers are wiped with DNA Away before placed in the ancient DNA lab. Personnel are restricted in their movement and are restricted from entering the ancient DNA after being in a contemporary DNA laboratory. A database containing mitochondrial control region sequence is maintained of all personnel working in the MMAL and of any personnel who may have come into contact with the human remains prior to DNA analysis.

Contamination controls were used with every DNA extraction and PCR setup in order to detect any contamination. Also, series of negative controls are routinely performed in the ancient DNA lab. DNA was extracted, the HVSI of the sample was sequenced and genotyped via RFLP for haplogroup diagnostic markers for mitochondrial haplogroups A and D using the same protocol as in Malhi et al. [24]. These samples were extracted at least twice for each sample in the Malhi lab. To provide additional confirmation that the DNA results were not derived from lab specific contaminants, samples from Ancient 939 and Ancient 938 were sent to the ancient DNA lab at Jilin University in China. At Jilin University, DNA from each sample was extracted twice and the results were confirmed by sequencing the HVSI portion of the mitochondrial genome and by RFLP for haplogroups A and D following Li et al. [52]. In addition, tooth sample from Ancient 160a was sent to the ancient DNA lab at Washington State University (WSU), Pullman. A 66 mg portion of the root was removed from the whole and submerged in 6% sodium hypochlorite (full strength Clorox bleach) for 4 min. The sample was then rinsed twice with DNA-free ddH_2_O and moved to 1.5 mL tubes to which 500 µL of EDTA (pH 8.0) was added. This was accompanied by an extraction negative control of 500 µL of EDTA, to which no sample was added. The tubes were incubated with agitation at room temperature for 48 hours. Sixty units of proteinase K (Biobasic) were added to the tubes and incubated at ∼65°C for 3 hours. The volumes were transferred to 5 mL Falcon tubes, to which 750 µL of 2% celite in 6 M guanidine HCl and 250 µL of 6 M guanidine HCl were added. The tubes were vortexed numerous times over a 2-minute period. These mixtures were pulled across Promega Wizard® Minicolumns using Luer-Lok syringes and a vacuum manifold. The silica pellets were rinsed by pulling 3 mL of 80% isopropanol across the columns. Residual isopropanol was removed from the columns by centrifugation in 1.5 mL tubes at 10,000 g for 2 min. The columns were moved to new 1.5 mL tubes and 50 µL of 65°C DNA-free ddH_2_O was added to the column and left for 3 minutes prior to centrifugation at 10,000 g for 30 s, and this step was repeated again resulting in 100 µL of extracted DNA. Markers definitive of Native American mitochondrial DNA haplogroups A–D were screened and D-loops 1–4 directly sequenced (corresponding to nps 16011–16382) according to Kemp et al. [23].

## Supporting Information

Figure S1 **DNA damage signatures.** The graphs demonstrate an excess of purines at the genomic coordinates located right before the sequence start. This pattern is indicative of ancient DNA, where post-mortem depurination occurs followed by a subsequent fragmentation (Briggs, Stenzel, Johnson, *et al.* 2007). The Perl script, mapDamage 0.36, measured this pattern globally across the aligned SAM file.
(DOCX)
