# Supplementary material for: Ancient DNA Analysis of Mid-Holocene Individuals from the Northwest Coast of North America Reveals Different Evolutionary Paths for Mitogenomes
Source: PLoS One. 2013 Jul 3;8(7):e66948. doi: 10.1371/journal.pone.0066948 (PMC3700925; doi:10.1371/journal.pone.0066948)
Supplement: Figure S1 — DNA damage signatures. The graphs demonstrate an excess of purines at the genomic coordinates located right before the sequence start. This pattern is indicative of ancient DNA, where post-mortem depurination occurs followed by a subsequent fragmentation (Briggs, Stenzel, Johnson, et al. 2007). The Perl script, mapDamage 0.36, measured this pattern globally across the aligned SAM file. (DOCX) [file pone.0066948.s001.docx]

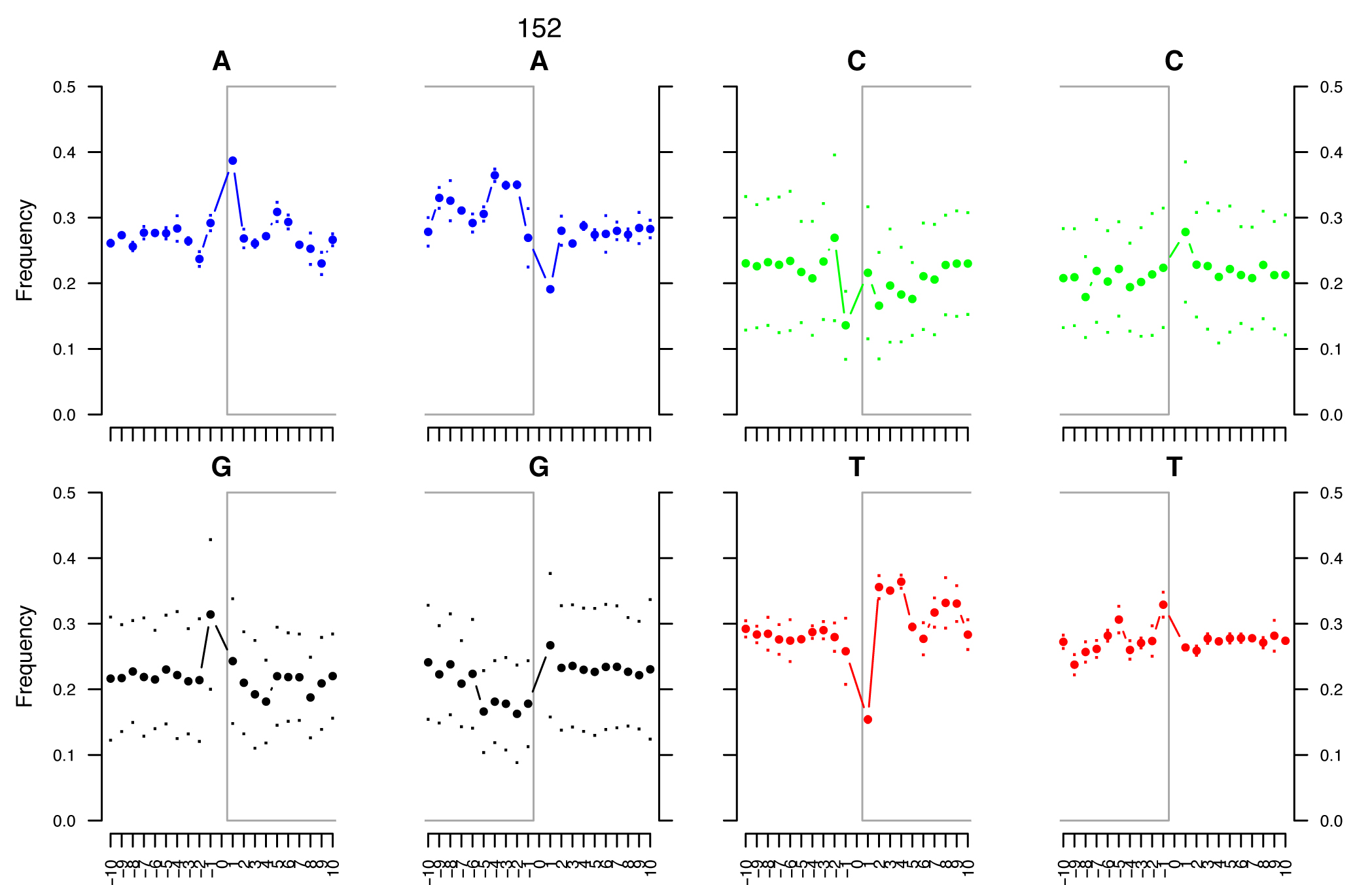

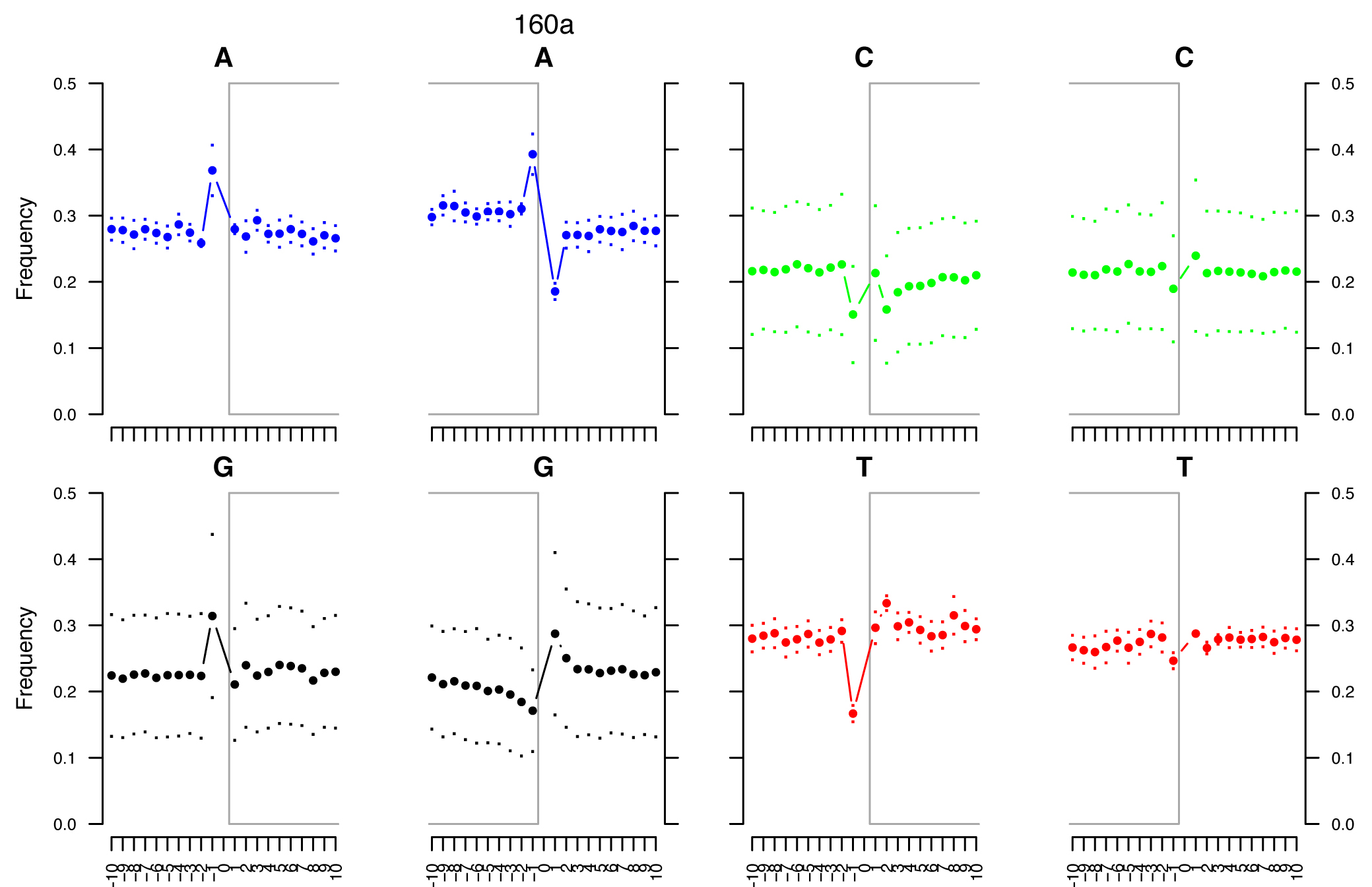


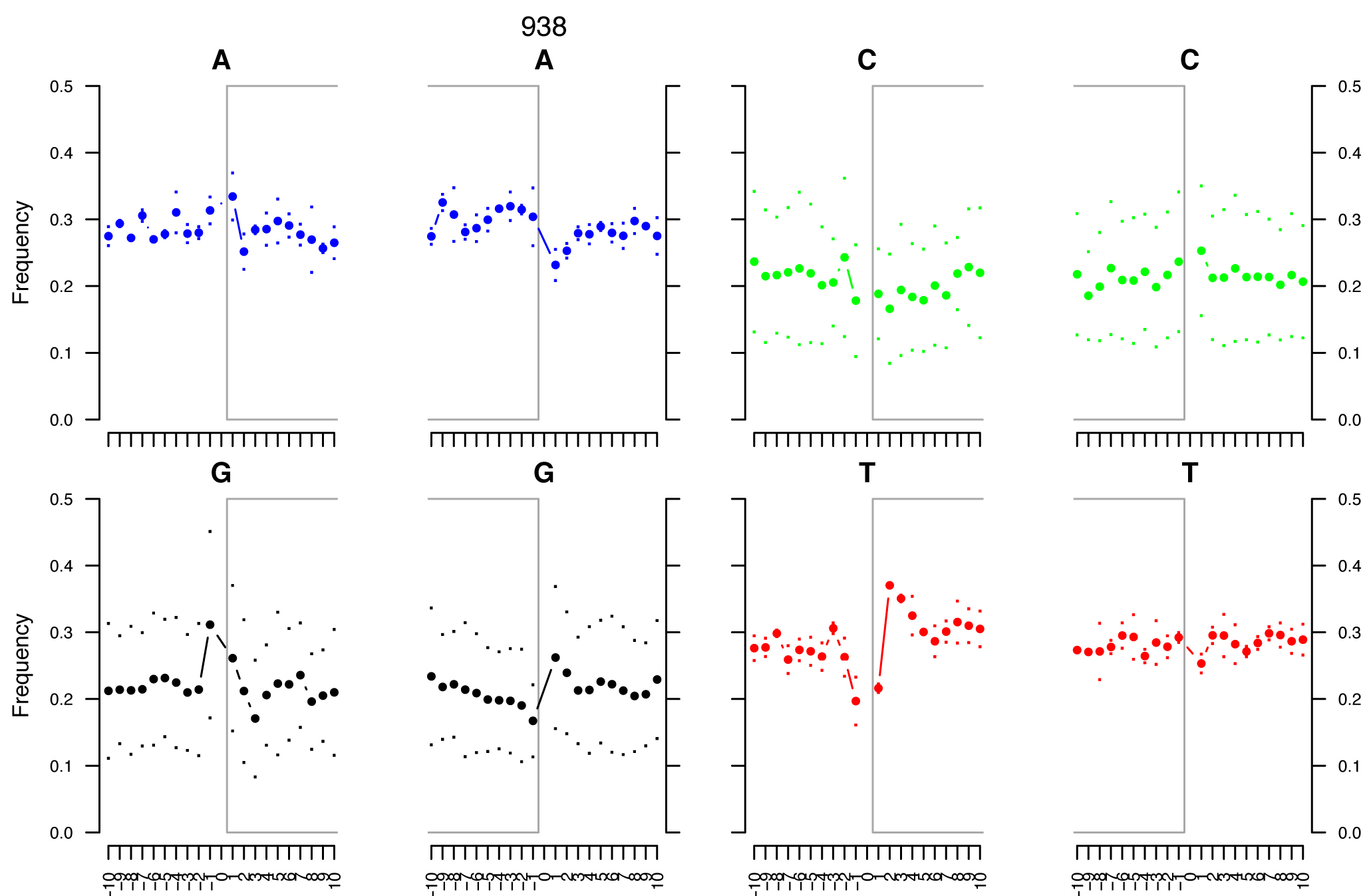

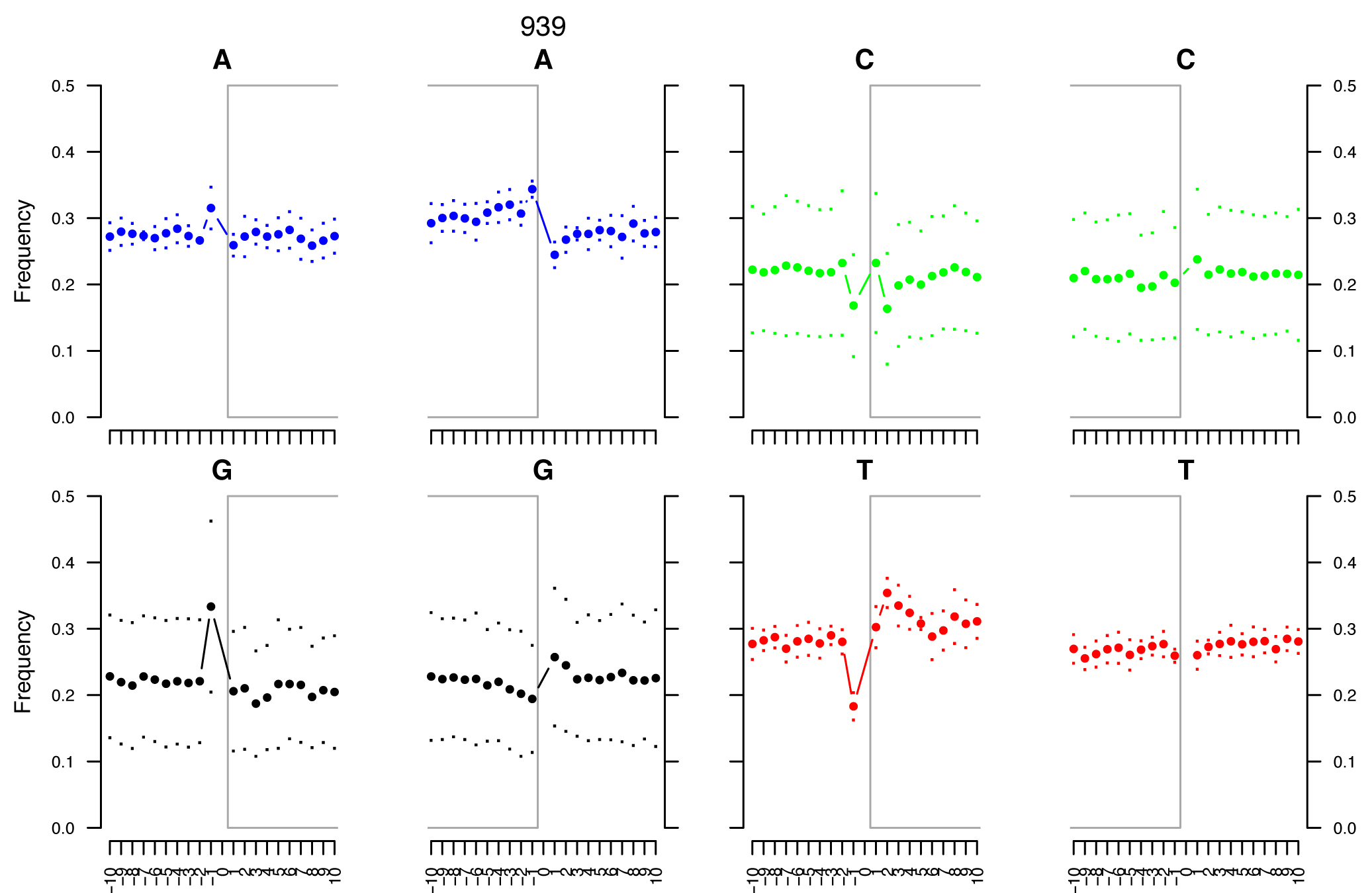


**Supplemental Figure 1. DNA Damage Signatures.** The above graphs demonstrate an excess of purines at the genomic coordinates located right before the sequence start. This pattern is indicative of ancient DNA, where post-mortem depurination occurs followed by a subsequent fragmentation (Briggs, Stenzel, Johnson, *et al.* 2007). The Perl script, mapDamage 0.36, measured this pattern globally across the aligned SAM file.
